# Salivary protein candidates for biomarkers of oral disorders in people with a crack cocaine use disorder

**DOI:** 10.1590/1678-7757-2022-0480

**Published:** 2023-05-15

**Authors:** Cassiano Lima CHAIBEN, Nayara Flores MACEDO, Thiago Beltrami Dias BATISTA, Carlos Antonio Schaffer PENTEADO, Talita M. O. VENTURA, Aline DIONIZIO, Paulo Henrique Couto SOUZA, Marília Afonso Rabelo BUZALAF, Luciana Reis AZEVEDO-ALANIS

**Affiliations:** 1 Pontifícia Universidade Católica do Paraná Escola de Ciências da Vida Programa de Pós-graduação em Odontologia Curitiba PR Brasil Pontifícia Universidade Católica do Paraná, Escola de Ciências da Vida, Programa de Pós-graduação em Odontologia, Curitiba, PR, Brasil.; 2 Universidade de São Paulo Faculdade de Odontologia de Bauru Departamento de Ciências Básicas Bauru SP Brasil Universidade de São Paulo, Faculdade de Odontologia de Bauru, Departamento de Ciências Básicas, Bauru, SP, Brasil.

**Keywords:** Salivary proteins and peptide, Proteomics, Biomarkers, Crack cocaine

## Abstract

The use of cocaine and its main derivative, crack, can cause some systemic effects that may lead to the development of some oral disorders.

**Objective:**

To assess the oral health of people with a crack cocaine use disorder and identify salivary protein candidates for biomarkers of oral disorders.

**Methodology:**

A total of 40 volunteers hospitalized for rehabilitation for crack cocaine addiction were enrolled; nine were randomly selected for proteomic analysis. Intraoral examination, report of DMFT, gingival and plaque index, xerostomia, and non-stimulated saliva collection were performed. A list of proteins identified was generated from the UniProt database and manually revised.

**Results:**

The mean age (n=40) was 32 (±8.88; 18–51) years; the mean DMFT index was 16±7.70; the mean plaque and gingival index were 2.07±0.65 and 2.12±0.64, respectively; and 20 (50%) volunteers reported xerostomia. We identified 305 salivary proteins (n=9), of which 23 were classified as candidate for biomarkers associated with 14 oral disorders. The highest number of candidates for biomarkers was associated with carcinoma of head and neck (n=7) and nasopharyngeal carcinoma (n=7), followed by periodontitis (n=6).

**Conclusions:**

People with a crack cocaine use disorder had an increased risk of dental caries and gingival inflammation; less than half had oral mucosal alterations, and half experienced xerostomia. As possible biomarkers for 14 oral disorders, 23 salivary proteins were identified. Oral cancer and periodontal disease were the most often associated disorders with biomarkers.

## Introduction

The use of cocaine and its main derivative, crack, has become a huge public health concern worldwide due to the medical, psychological, and social issues associated with its abuse. The estimation of people using cocaine worldwide is 17 to 20 million.^1^

The systemic effects of crack cocaine addiction include cardiovascular, neurological, respiratory, pulmonary, and gastrointestinal complications.^2^ People with a cocaine use disorder have a high prevalence of anorexia and malnutrition, which may lead to the development of secondary lesions in the oral mucosa,^3,4^ with glossalgia, angular cheilitis, and other forms of candidiasis being the most common.^5-7^ Several studies have reported alteration in taste perception in people with a crack cocaine use disorder.^8^ Cytological examinations have shown alterations in their mucosal cells, suggesting DNA damage, inflammation, enucleated cell enlargement, and in the number of nucleolar organizer regions. In addition to inflammation, these results suggest intense tissue proliferation and differentiation, disregarding drug aggression.^9-12^

Human tissues and biological fluids contain proteins and other molecules that can be used as biomarkers. The comparative and mass spectrometry-based proteomic analysis of body fluids can be particularly instrumental for the targeted identification of novel protein biomarkers with pathological relevance.^13^ A biomarker can be defined as an indicator of physiological process, disease, or response to a therapeutic agent.^14,15^Although the presence or change in concentration of a biomarker is detected during a disease process, it does not guarantee disease occurrence. Careful evaluation of clinical parameters in the patient should be considered.^16^

Saliva is an excellent diagnostic fluid due to its noninvasive method of collection;^17,18^ the proximity to tumors of the head and neck offers great potential for biomarker detection in saliva. Proteomic analysis of this fluid allows identification of proteins that function as early diagnostic tools or prognostic markers for diseases.^17^ Salivary proteomics research was used to characterize the proteome profile of alcoholics and smokers^19^and to identify possible biomarkers of oral disorders in this population.^20^ Nonetheless, there are only a few research on the protein profile on the saliva of people with a crack cocaine use disorder.^21^

Thus, this study aimed to evaluate the oral health condition of people with a crack cocaine use disorder and to identify salivary protein candidates for biomarkers of oral disorders.

## Methodology

The local Institutional Review Board approved the experimental protocol of this study (approval No. 1825659).

Volunteers were recruited from the Instituto de Pesquisa e Tratamento do Álcool (IPTA - Campo Largo, Paraná, Brazil). Men above 18 years and who were undergoing treatment for crack cocaine addiction with a maximum of 15 days of abstinence^8^ were enrolled for the study. Participants with systemic disorders or acute infection symptoms, using antibiotics or anti-inflammatory drugs,^19-21^ and with oral mucosal injuries were excluded. An informed consent form was signed before any procedure by all participants.

The participants were questioned about their socioeconomic background, drug usage history (both licit and illicit), overall health, use of medications, and oral hygiene. Next, extraoral clinical examinations and intraoral assessments were conducted in both natural and artificial light.^20^ The presence or absence of xerostomia was assessed by the question “Do you have a dry mouth sensation?”; xerostomia was considered present if the participant answered “yes”. In cases of oral or gingival mucosal alterations or dental caries the subjects were referred to the dental clinic at the Pontifical Catholic University of Paraná. The decayed, missing, and filled teeth (DMFT) index, gingival index (GI), and plaque index (PI) were evaluated.^20^

A total of 40 volunteers were included in the study. Among them, nine were randomly selected for salivary proteomics analysis.^19-22^

### Proteomic Analysis

The collection of saliva was performed as described in previous studies.^19-23^ Proteins were extracted separately from each sample and biological triplicates were prepared immediately after extraction. Peptide identification was performed on nanoACQUITY UPLC Xevo Q-TOF MS system (Waters, Milford, MA, USA), according to other studies.^19-22^

The ion count technique included in the ProteinLynx Global Server program (PLGS) version 3.03 (Waters, Milford, MA) was used to identify salivary proteins. The results were obtained by searching the Homo sapiens database in the UniProt catalogue (Universal Protein Resource), version August 2017 (http://www.uniprot.org). All pooled samples were analyzed in triplicate.^19-22,24^

From the results generated by the mass spectrometer, a manual revision of the protein list was performed.^20-25^ Subsequently, the genes of origin of each protein were identified and used to search for protein candidates for biomarkers in the search tool IBI-IMIM (Database of disease-related biomarkers).^20,25,26^

All study reports that investigated the possible association of proteins with oral disorders were manually reviewed to ensure that they effectively investigated the association between biomarkers and oral disorders cited in the IBI-IMIM search tool. To compile a final list of proteins that have been searched for putative association with oral disorders, articles in which the main protein, disorder, or both were not described were excluded.^20^

## Results

The sample comprised 40 men with the mean age of 32 (±8.88; 18–51) years and mean DMFT of 16 (±7.70; 0–26). The most frequent clinical changes were leukoedema, actinic cheilitis, and smoker’s melanosis; 20 participants (50%) reported xerostomia. Table 1 shows all clinical changes along with the drug use profile, PI, and GI of the participants.

**Table 1:** Clinical changes, oral hygiene pattern, and drug use profile of all participants (n=40) and the participants selected for salivary proteomics analysis (n=9)

Oral changes	n=40	n=9
	N (%)	N (%)
Leukoedema	14 (35)	3 (33.33)
Actinic Cheilitis	12 (30)	2 (22.22)
Smoker’s melanosis	6 (15)	1 (11.11)
Reactional keratosis	3 (7.5)	0
Nicotinic stomatitis	1 (2.5)	0
Chronic hyperplastic candidiasis	1 (2.5)	0
	**Average ± Standard deviation**	
Age (years)	32±8.88	36.39±7.78
DMFT	16±7.70	13.75±8.10
Plaque index	2.07±0.65	2.12±0.64
Gingival index	2.12±0.64	1.62±0.74
Other	**N (%)**	**N (%)**
Tobacco	40 (100)	9 (100)
Alcohol	40 (100)	9 (100)
Reported xerostomia	20 (50)	6 (66.66)
Non-stimulated salivary flow	0.77 mL/min	0.74 mL/min

The nine individuals selected for salivary proteomics analysis had a mean age of 36.39 (±7.78; 28–51) years. A total of 305 salivary proteins were identified by proteomic analysis. From that, 32 proteins were found to be candidate for biomarkers of oral disorders; 19 different oral disorders were identified.

The most frequent oral disorders were “hereditary gingivitis fibromatosis” (11 biomarkers), “pleomorphic adenoma” (9 biomarkers), and “carcinoma of head and neck” (7 biomarkers), and “nasopharyngeal carcinoma” (7 biomarkers) (Table 2).

**Table 2:** Oral disorders, protein code (accession number), and number of candidates for biomarkers before manual revision

Oral disorder	Accession number	n
Hereditary gingival fibromatosis	P18827, P02042, P69892, P68871, P02768, P49327, P06276, O00533, Q12802, P06733, B6EC88	11
Pleomorphic adenoma of salivary gland	P02768, O00533, H7BZ45, P04406, P09104, H0YJZ5, P06702, B6EC88, P05109	9
Carcinoma of head and neck	P18827, P01034, Q8TAX7, A0A1W2PQJ4, P23528, H0YJZ5, B6EC88	7
Nasopharyngeal carcinoma	P18827, Q15582, P02768, P61626, P01833, P06733, B6EC88	7
Periodontitis	P04746, P01034, P02768, P13686, P06702, B6EC88	6
Periodontal disease	P01034, P02768, P61626, B6EC88, P05109	5
Rhabdomyosarcoma, alveolar	P01034, P02768, P02686, P13686	4
Kaposi's sarcoma (clinical)	P18827, P12273, P23528	3
Dental caries	P61626, B6EC88, A0A0U1RQT3	3
Ameloblastoma	P18827, O00533	2
Squamous cell carcinoma of tongue	P18827, P49327	2
Gingivitis	P18827, P01034	2
Acquired absence of teeth	P01034	1
Tooth disorder	P02768	1
Leukoplakia	P02768	1
Candidiasis of mouth	P31025	1
Root resorption	P13686	1
Hyposecretion of salivary gland	A0A1B0GV13	1
Periodontal pocket	B6EC88	1

The most frequent terms related to changes in denture-supporting tissues were “periodontitis” (6 biomarkers), “periodontal disease” (5 biomarkers), “gingivitis” (2 biomarkers), “periodontal pocket” (1 biomarker), and “acquired absence of teeth” (1 biomarker). Alteration in three terms related to teeth were associated with one biomarker each: dental caries, root resorption, and dental disorder. Table 2 shows all oral disorders identified with their respective biomarkers.

After manual review, all articles related to “pleomorphic adenoma of salivary gland,” which focused on other types of adenomas, were excluded since the biomarkers were not related to oral disorders indicated by the database. After exclusion, a total of 23 salivary protein as candidates for biomarkers associated with 14 oral disorders were identified (Table 3).

**Table 3:** Oral disorders, protein code (accession number), and number of candidates for biomarkers after manual revision

Oral disorder	Accession number	n
Carcinoma of head and neck	P18827, P01034, Q8TAX7, A0A1W2PQJ4, P23528, H0YJZ5, B6EC88	7
Nasopharyngeal carcinoma	P18827, Q15582, P02768, P61626, P01833, P06733, B6EC88	7
Periodontitis	P04746, P01034, P02768, P13686, P06702, B6EC88	6
Periodontal disease	P01034, P02768, B6EC88, P05109, P06702	5
Hereditary gingival fibromatosis	P02042, P69892, P68871, P49327	4
Kaposi's sarcoma (clinical)	P18827, P12273, P23528	3
Ameloblastoma	P18827, O00533	2
Squamous cell carcinoma of tongue	P18827, P49327	2
Gingivitis	P18827, P01034	2
Tooth disorder	P02768	1
Leukoplakia	P02768	1
Root resorption	P13686	1
Hyposecretion of salivary gland	A0A1B0GV13	1
Periodontal pocket	B6EC88	1

## Discussion

In Brazil, among illicit substances, the use of cocaine and crack by adults are ranked second and third, respectively.^27^ The use of crack cocaine is associated with a variety of systemic^4,28^ and oral diseases.^29-30^ Salivary proteomics analysis, along with the identification of protein candidates for biomarkers of these disorders, may assist in the early diagnosis and framing health policies for both people with a crack cocaine use disorder and those who previously used it in an unprecedent manner.

Regarding oral condition of people with a crack cocaine use disorder, this study reported high DMFT index, in addition to the presence of dental plaque and gingival inflammation, which agrees with other studies conducted on similar populations and age groups.^31,32^ The study sample presented a gingival index (score 2) indicating moderate inflammation (score 2) without a large accumulation of dental biofilm. These clinical findings may explain the presence of salivary protein biomarkers of gingivitis, periodontitis, periodontal disease, and periodontal pocket in the tested sample.

In this study, a biomarker for hyposalivation (Spectrin alpha - A0A1B0GV13) was identified by salivary proteomics analysis, which agrees with a high percentage of the sample (50%) reporting xerostomia; however, a study that indicated similar correlation in the IBI-IMIM search tool was performed in patients with Sjögren’s syndrome.^33^ Other studies showed a significant reduction in salivary pH, but no changes in salivary flow and buffer capacity of the saliva collected from people with a crack cocaine use disorder.^29,32^ The xerostomia reported by the volunteers in this study might be associated with the use of medications for the treatment of abstinence and not directly to the use of crack cocaine.

Hereditary gingival fibromatosis (HGF) was found to be associated with four biomarkers according to two previous studies.^34,35^ According to Almeida et al.^34^ (2005), the production of Fatty Acid Synthase (FAS) activity was greater in patients with HGF than in others, and inhibition of FAS caused a significant reduction in the production of fibroblasts, even in healthy patients. Conversely, FAS was found to be related to the growth factor of several tumors and considered an indicator of prognosis in neoplasia.^36^ This controversy may suggest that FAS is a biomarker related to tissue proliferation and may not be directly involved in the production of fibroblasts in HGF, as proposed by Almeida, et al.^34^(2005).

The albumin biomarker (P02768) was related to leukoplakia, as reported in two previous studies.^37,38^ Tobacco use is associated with a decrease in serum albumin concentration, as this protein is reduced in patients diagnosed with leukoplakia. Leukoplakia is one of the most common potentially malignant lesions, with a relatively high risk of transformation into squamous cell carcinoma.^38^ Alcohol and tobacco are the main risk factors for this lesion,^39^ and the use of both was frequent in our study sample. Furthermore, tobacco use was also associated with smoker’s melanosis and nicotine stomatitis in this study.

Actinic cheilitis, which is considered a potentially malignant lesion caused by excessive ultraviolet radiation exposure, was diagnosed in our sample. Moreover, carcinoma of head and neck was associated with seven biomarkers, and 10 articles have reported on diagnostic, prognostic, and new treatment targets for this disease. The salivary proteomics analysis of patients with carcinoma of head and neck showed high levels of proteins associated with tumor progression and metastasis, particularly cofilin-1 (P23528).^17^ This biomarker is increased in tissue areas surrounding the margin of safety from samples of oral carcinoma.^17^ The biomarker syndecan-1 (P18827), also found in our sample, was found to be associated with the degree of histological differentiation of carcinoma.^14^Reduced syndecan-1 expression was linked to a low histologic grade of differentiation and a poor outcome in patients with head and neck neoplasms treated with surgery and postoperative radiation.^40^ However, an analogy of these studies with our sample is difficult since the age of the population is either different or not mentioned. Moreover, people with a crack cocaine use disorder tend to have premature mortality due to other factors, such as complications of HIV/AIDS or other behaviors.^27^

Proteomic analysis allows for the identification of many biomarkers in a short time in complex biological matrices such as saliva. Thus, higher selectivity, specificity, and sensitivity could be obtained by using techniques to perform sequence analysis of the tryptic peptides, providing supplementary unique data on the mass and structure of the selected required component. Therefore, standardization is required in the preanalytical phase,^22^ and a step of peptide separation based on size following total tryptic digestion can help isolate a large peptide population. Furthermore, search tools should be incorporated into databases by using programs, although some may have limitations. In this study, IBI-IMIM, despite being an excellent tool, presented several incorrect associations between biomarkers and pathological processes.

Upon manual review, we found that some biomarkers were associated with other diseases or were mentioned in the articles but not related to the target process of the study. This suggests that while researchers can use these search tools for pre-analysis, the results should be critically and manually checked before being included in their review. Another limitation of this study is its descriptive design, which does not allow for verification whether a patient will develop a disorder related to the identified biomarker; a larger sample size and a prospective study design would provide answers to these questions. Finally, the exclusion of women subjects is another limitation of this study. Since hormonal variations could affect salivary protein secretion in women^19^, and only male patients over 18 years are attended at IPTA, only men were included in this study.

## Conclusion

People with a crack cocaine use disorder had an increased risk of dental caries and gingival inflammation. Less than half had alterations in their oral mucosa, while half experienced xerostomia. A total of 23 salivary proteins were identified as candidates for biomarkers of 14 oral disorders. Oral cancer and periodontal diseases were the most often associated disorders with biomarkers. Proteomic analysis may play a crucial role in understanding alterations in the salivary protein profile associated with crack cocaine use and may serve as a tool for accurate and early detection of disorders affecting the head and neck region.
